# *Tripterygium wilfordii* Glycosides Upregulate the New Anti-Inflammatory Cytokine IL-37 through ERK1/2 and p38 MAPK Signal Pathways

**DOI:** 10.1155/2017/9148523

**Published:** 2017-12-18

**Authors:** Sen Wang, Rumeng Li, Suhui He, Lingge He, Hang Zhao, Xiaohong Deng, Zhangquan Chen

**Affiliations:** Key Laboratory for Medical Molecular Diagnostic of Guangdong Province, Guangdong Medical University, Dongguan, Guangdong Province 523808, China

## Abstract

As a Chinese traditional patent medicine,* Tripterygium wilfordii* glycosides (TWG) have been approved by the China State Food and Drug Administration (Z32021007) for autoimmune and inflammatory diseases. Application of TWG leads to significant decrease of the inflammatory cytokines, such as IL-6, IL-1*β*, and TNF-*α*. However, little is known whether TWG could regulate the anti-inflammatory cytokines and what the mechanism is. Here, we found that TWG could induce the upregulation of IL-37 which is a new anti-inflammatory cytokine. Furthermore, the inhibitors of ERK1/2 and/or p38 MAPK pathways suppressed IL-37 expression induced by TWG, indicating that the two pathways took part in this process. In conclusion, TWG could upregulate the anti-inflammatory cytokine IL-37 and ERK1/2 and p38 MAPK signal pathways were involved in the upregulation of IL-37 induced by TWG. The results showed that TWG had a potent activity on promoting the expression of IL-37, a new anti-inflammatory cytokine, which help further understanding the anti-inflammatory mechanism for the clinical application of TWG in therapy of diseases.

## 1. Introduction

During the years,* Tripterygium wilfordii* glycosides (TWG) have been demonstrated to be a powerful anti-inflammatory and immune modulatory drug [1, 2]. TWG is a stable glycoside extracted from* Tripterygium wilfordii* Hook F (TwHF). In China, America, Korea, Japan, and other countries, TwHF is known as “LeiGongTeng” for treating autoimmune diseases [1–4]. Thus, TWG from TwHF has been approved by the China State Food and Drug Administration (Z32021007) for the routine treatment of Crohn's disease [5], rheumatoid arthritis (RA) [3], and ulcerative colitis [6]. TWG tablets can be orally administered. After entering the body, TWG is absorbed by passive diffusion. The organ with highest drug concentration is liver. And TWG is excreted mainly through feces and urine [7]. Treatment with TWG leads to significant decrease of the inflammatory cytokines. In ulcerative colitis, dextran sulfate sodium-induced IL-6 expression was inhibited by triptolide, the main extract from TWG [6]. Another study found that TWG inhibited the inflammatory mediators in interleukin-1*β*-stimulated cells [8]. In another report, TWG could markedly reduce IL-6, IL-8, and TNF-*α* in serum of type II collagen-induced arthritis rats [9]. However, little is known whether TWG could regulate the anti-inflammatory cytokines and what the mechanism is. This problem is quite important to understand fully the treatment mechanism of TWG for autoimmune and inflammatory diseases.

Besides the inhibition of the expression of inflammatory cytokines, the mechanism of anti-inflammatory drugs often includes the effects on anti-inflammatory cytokines. Until recently, few attempts have been done for TWG on the influence of anti-inflammatory molecules. IL-37 is a newly named member of IL-1 family with powerful anti-inflammatory function. IL-37 contains five subtypes, IL-37a, b, c, d, and e, and IL-37 usually refers to IL-37b which is the largest subtype and shows important bioactive function. This gene is found to be expressed in a variety of tissues and cells, such as monocytes, natural killer (NK) cells, and epithelial cells [10, 11]. Here, we aim to discuss whether TWG could regulate a new anti-inflammatory cytokine, IL-37, and to elucidate the related mechanism. Researchers have found that IL-37 mediates a negative feedback mechanism to curb the excessive inflammation [10, 12, 13]. IL-37 could repress the proinflammatory cytokines expression, including IL-6, TNF-*α*, and IL-1*β* to reduce the inflammation response [10, 14–16]. Most importantly, IL-37 was also involved in some inflammatory diseases that could be treated with TWG [17, 18], suggesting that TWG might have influenced IL-37 in those diseases. IL-37 expression has been found in human monocytes and human cell lines (such as THP-1, U937, A431, IMTLH, KG-1, HL60, HPT-4, and NHDC). THP-1 is a suitable cell model for IL-37 research in many reports including the study which renamed IL-37 in 2010 in Nature Immunology [10]. So THP-1 cells were chosen in this study. The THP-1 cellular model for IL-37 expression was used as previously described [10]. We found that TWG upregulated IL-37 expression in THP-1 cells. Furthermore, ERK1/2 and MAPK were involved in the regulation.

## 2. Materials and Methods

### 2.1. Reagents and Cell

Anti-IL-37 monoclonal antibody, Goat Anti-Mouse IgG H&L (FITC), and Mouse IgG1 were purchased from Abcam Corporation (Cambridge, UK). TWG (10 mg/tablet, Approval number Z35020431) was obtained in the form of tablets (*Tripterygium wilfordii* glycoside tablets) from Fujian Huitian Bio-pharma Co., Ltd. (GMP certificated, Fujian, China). The tablets were dissolved in the incubation medium at a final concentration of 5 mg mL^−1^ as stock solution. Inhibitor of extracellular signal-regulated kinase 1 (ERK1)/ERK2, U0126, and inhibitor of p38 MAPK, SB 203580, were from Cell Signaling Technology (Beverly, MA, USA). RNAiso Plus, Prime Script™ RT Master Mix and SYBR® Premix Ex Taq (Perfect Real Time) were from Takara Biotechnology Co., Ltd. (Dalian, China). Primers were synthesized by Sangon Biotech Co., Ltd. (Shanghai, China). eBioscience Fixation/Permeabilization Concentrate and Permeabilization Buffer (10×) were from eBioscience Inc. (San Diego, CA, USA). Dimethyl sulfoxide (DMSO) and phorbol-12-myristate 13-acetate (PMA) were from Sigma (St. Louis. MO, USA). THP-1 cell line was obtained from the Cell bank of Chinese Academy of Sciences, Shanghai, China. Cells culture reagents including 1640 medium and fetal bovine serum (FBS) were purchased from HyClone (Longan, UT, USA).

### 2.2. Cell Culture and Drugs Stimulation

THP-1 cells were maintained in RPMI-1640 medium at 37°C, 5% CO_2_. The 1640 medium contained 10% fetal bovine serum (FBS), 100 U mL^−1^ penicillin, and 0.1 mg mL^−1^ streptomycin. Then, 1 × 10^6^ mL^−1^ cells were planted in 48-well culture plates and were treated with 50 ng mL^−1^ PMA for differentiation for 48 h [9]. The plastic-adherent cells were washed twice with sterile Dulbecco's phosphate-buffered saline (PBS) and incubated with fresh RPMI 1640 medium containing 0.5% FBS for 18 h. On the basis of previous reports, TWG concentrations from 0 to 15 *μ*g mL^−1^ were adopted to determine concentration-dependent effects of TWG on expression of IL-37 in the preliminary test. After incubation for different periods, the prepared cells were collected for analysis.

### 2.3. Quantitative Real-Time PCR

Total RNA was extracted by a Takara RNAiso Plus Kit. Then, cDNA was synthesized through a Prime Script RT Master Mix Kit. A SYBR Premix Ex Taq TM kit was used for Real-time quantitative PCR which was carried out on the ABI prism 7700 Sequence Detection System (Perkin Applied System, Foster City, CA, USA). The reaction was performed in 25 *μ*L reaction buffer. The reaction buffer was mixed with 12.5 *μ*L of 2× SYBR green Master Mix, 1 *μ*L cDNA, and 300 nM primer. Here, GAPDH primers for PCR were as follows: F (ACCCAGAAGACTGTGGATGG) and R (TTCTAGACGGCAGGTCAGGT). Here, the subtype of IL-37 is IL-37b. The specific primers for real-time PCR were as follows: F (TTAGAAGACCCGGCTGGAAGCC) and R (AGATCTCTGGGCGTATGTAGT). PCR cycling conditions were 95°C for 2 min (at 95°C for 5 s, 60°C for 30 s, and 72°C for 30 s) for 40 cycles. The thermal dissociation protocol was for the detection of the PCR product. Each experiment was done in triplicate.

### 2.4. Flow Cytometry Analysis of Intracellular IL-37

Cells were firstly incubated with fresh RPMI 1640 medium containing 0.5% FBS for 18 h. Then, after pretreatment with TWG for 16 h, the cells were collected and washed with ice cold staining buffer and were resuspended at 1.0 × 10^9^ L^−1^. These cells were fixed with 100 *μ*L Fixation/Permeabilization working buffers and incubated for 20 minutes at room temperature in the dark. Next, 2 mL 1× Permeabilization working buffer was added to each tube. After washing twice, the cell pellets were resuspended with 100 *μ*L 1x Permeabilization buffer which contained 10% normal goat serum for serum blockage and were incubated for 1 h in the dark at room temperature. Then, cells were incubated with 1 *μ*L of IL-37 monoclonal antibody or mouse IgG1 isotype control antibody for 1 h. Cells were washed with Permeabilization working buffer and incubated with the secondary antibody at 1/500 dilution for 30–60 min in the dark. Finally, the stained cells were acquired on the flow cytometer and the data were analyzed by BD FACSDIva software.

### 2.5. Analysis of Signal-Transduction Pathways

To discuss the mechanism involved in the over expression of IL-37 induced by TWG, the specific inhibitor for p38 MAPK or ERK1/2 pathway was used, respectively. After being pretreated with p38 MAPK inhibitor, SB203580 (10 *μ*mol L^−1^), or ERK1/2 inhibitor, U0126 (5 *μ*mol L^−1^), for 30 min, macrophages from THP-1 cells were treated with TWG. The cells without pretreatment with SB203580 or U0126 were chosen as controls. After the drug stimulation for 12 h, the cells were collected for RNA extraction for real-time PCR. After drug stimulation for 16 h the cells were collected for flow cytometry analysis.

### 2.6. Statistics

All experiments were performed at three times. Data were expressed as means ± SD and were analyzed with one-way analysis of variance (ANOVA) utilizing the SPSS version 13.0. Different groups were considered to be statistically significant at *p* < 0.05.

## 3. Results

### 3.1. TWG Upregulated the Expression of IL-37 in THP-1 Cells

**Figure 1:**
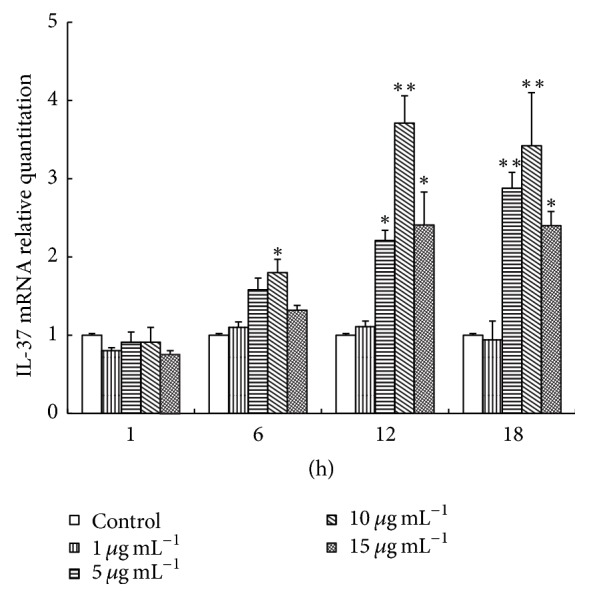
IL-37 mRNA induced by TWG in macrophages was detected by quantitative real-time PCR. TWG represents* Tripterygium wilfordii* glycosides. *∗* versus control, *p* < 0.05; *∗∗* versus control, *p* < 0.01.

Macrophages were from THP-1 cells treated with PMA [10]. Here, TWG concentrations were 1 *μ*gmL^−1^, 5 *μ*gmL^−1^, 10 *μ*gmL^−1^, and 15 *μ*gmL^−1^ for pretreatment of cells. After drug stimulation, total RNA was extracted from macrophages for cDNA synthesis and IL-37 mRNA was detected by qPCR. The results were presented in Figure 1. At 1 h, IL-37 mRNA expression showed no obvious change for TWG at 1 *μ*gmL^−1^, 5 *μ*gmL^−1^, 10 *μ*gmL^−1^_,_ and 15 *μ*gmL^−1^. At 6 h, 12 h, and 18 h, 10 *μ*gmL^−1^ TWG showed the strongest capacity to enhance IL-37 expression. Furthermore, the levels of IL-37 expression elevated gradually with increasing incubation time from 1 h to 12 h in the presence of 10 *μ*g mL^−1^ of TWG. When time was over 12 h, the mRNA level began to decrease with further incubation, which indicated that the maximum IL-37 mRNA expression is reached.

**Figure 2:**
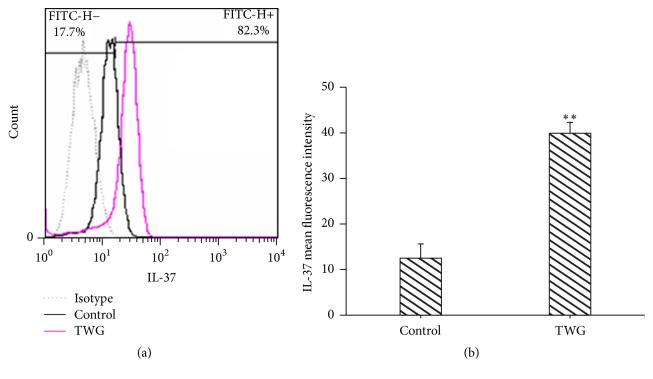
Effect of TWG on IL-37 protein in macrophages was examined by flow cytometry analysis. TWG represents* Tripterygium wilfordii* glycosides. *∗∗* versus control, *p* < 0.01.

In order to study more about the influence of TWG on expression of IL-37, IL-37 protein was examined by flow cytometry analysis. As shown in Figure 2, TWG upregulated the protein level of IL-37 in cells. IL-37 positive cells were approximately up to 82.3% in cells treated with 10 *μ*g mL^−1^ of TWG with 16 h incubation. Only 17.7% IL-37 positive cells were detected in THP-1 cells without TWG treatment (negative control) (Figure 2(a)). The mean fluorescence intensity (MFI) of cells treated with TWG was approximately 3.2-fold greater than that without TWG treatment (Figure 2(b)).

### 3.2. Effect of SB203580 and U0126 on TWG-Induced IL-37 Expression in THP-1 Cells

**Figure 3:**
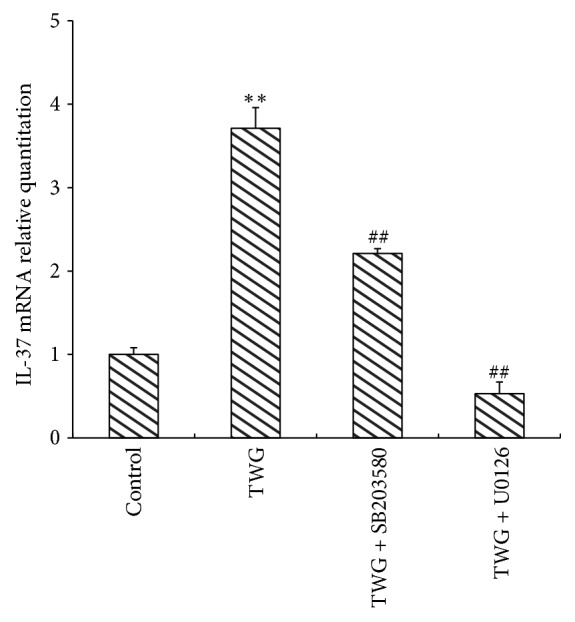
Effects of p38 inhibitor SB203580 and the ERK 1/2 inhibitor U0126 on TWG-induced mRNA expression of IL-37 were examined by real-time PCR in THP-1 cells. *∗∗* versus control, *p* < 0.01; ##  versus TWG, *p* < 0.01.

**Figure 4:**
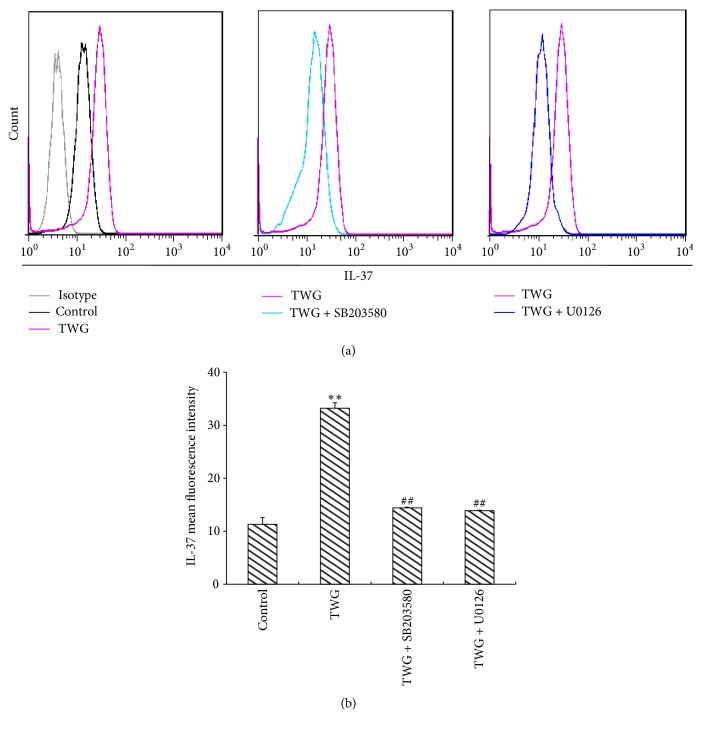
Effects of p38 MAPK inhibitor SB203580 and the ERK 1/2 inhibitor U0126 on the TWG-induced IL-37 protein were detected by flow cytometry in macrophages. TWG represents* Tripterygium wilfordii* glycosides. *∗∗* versus control, *p* < 0.01; ## versus TWG, *p* < 0.01.

In order to further elucidate how TWG induces the upregulation of IL-37, p38 MAPK and ERK1/2 signaling pathways were investigated. The cells were treated with SB203580 or U0126 for 30 min, respectively. Then the macrophages were incubated with TWG for 12 h. The expression of IL-37 was measured by real-time PCR (Figure 3) and flow cytometry (Figure 4). The results showed that SB203580 or U0126 decreased markedly the expression of IL-37 mRNA (Figure 3) and IL-37 protein (Figure 4). The results of qPCR showed that U0126 at 5 *μ*mol·L^−1^ almost completely abolished the expression of IL-37 mRNA induced by TWG in macrophages (Figure 3). The other inhibitor (SB203580) at 10 *μ*mol·L^−1^ reduced about 45% of IL-37 mRNA expression induced by TWG at 12 h (Figure 3).

As to the cytometry analysis, U0126 and SB203580 abolished most of IL-37 expression induced by TWG at 16 h of incubation in macrophages (Figure 4(a)). The mean fluorescence intensity (MFI) of cells treated with TWG + U0126 or TWG + SB203580 was approximately 50% of that of TWG group (Figure 4(b)).

## 4. Discussion

TWG is extracted from a traditional Chinese medicine herb,* Tripterygium wilfordii* Hook F (TwHF). TWG has been widely used in the treatment of inflammatory diseases [1–4, 19]. Growing evidences prove that TWG could inhibit expression of inflammatory cytokines, such as IL-6, IL-1*β*, and TNF-*α*, to treat related diseases [6, 8]. However, whether and how TWG influence the anti-inflammatory cytokines are still unclear. Interleukin 37 is one of the few anti-inflammatory cytokines in the interleukin family. An enormous amount of researches has confirmed that the proinflammatory cytokines, including IL-6, TNF-*α*, and IL-1*β*, play pivotal roles in the pathogenesis of the chronic inflammatory disorders [20, 21]. IL-37 also participates in diseases which could be treated by TWG, such as RA [17, 18]. Thus, we speculated that TWG could regulate IL-37 expression besides the inhibition of inflammatory cytokines. In addition, THP-1-derived macrophages have been reported to be a valid model system for anti-inflammatory drug screening [10]. Consequently, THP-1-derived macrophages were used to assess the effect of TwFH extracts on expression of IL-37. Here, we found that use of TWG upregulated significantly the mRNA and protein of IL-37. In light of the powerful anti-inflammatory function, this result might be a new mechanism for TWG to treat diseases.

To further discuss the mechanism involved in the upregulation of IL-37 induced by TWG, cell signaling pathways were investigated in this study. ERK1/2 signaling pathway and the MAPK signaling pathway have been identified as the major signaling pathways in inflammation [10]. TWG has been reported to influence ERK1/2 and MAPK signal pathways in diseases [20, 21]. Thus, we hypothesized that TWG might regulate ERK1/2 and MAPK to influence IL-37 expression. Our results showed that the p38 MAPK inhibitor (SB203580) and ERK1/2 inhibitor (U0126) could inhibit markedly the upregulation of IL-37 induced by TWG. It should be noted that the results could not be used to determine that p38 MAPK and ERK1/2 are the only pathways for TWG-induced IL-37. However, this paper does suggest that ERK1/2 and p38 MAPK pathways might be involved in the upregulation of IL-37 induced by TWG. In addition, in the work described by Imaeda et al. [14], NF-kB and AP-1 are involved in the induction of IL-37 by TNF-*α*. However, TNF-*α* is an inflammatory mediator while TWG is an anti-inflammation drug. In the work described by Sylvester et al. [22], a* Tripterygium* extract was found to inhibit the activity of NF-kB and AP-1. Thus, it seems that MAPK and ERK signals are more possible to take part in the induction of IL-37 by TWG. And the results did show that MAPK and ERK signals were involved in the TWG-induced IL-37 elevation. Thus, our study indicated a new possible mechanism for TWG to curb inflammation.

## 5. Conclusion

In this study, we found that TWG could induce the upregulation of IL-37 and the ERK1/2 and/or P38 MAPK pathways were involved in this process. Thus, our study indicated a new possible mechanism for TWG to curb inflammation.

## Additional Points

*Highlights*.* Tripterygium wilfordii* glycosides upregulated the anti-inflammatory cytokine IL-37; ERK1/2 and p38 MAPK signal pathways were involved in the upregulation of IL-37 induced by TWG. We showed a new anti-inflammatory mechanism for* Tripterygium wilfordii* glycosides.

## Supplementary Materials

Supplementary Materials IL-37 is one of the few anti-inflammatory cytokines. TWG, a stable glycoside extracted from* Tripterygium wilfordii* Hook F (TwHF), is a powerful anti-inflammatory and immune modulatory drug. In this study, it is found that TWG could upregulate IL-37, and ERK1/2 and p38 MAPK pathways might be involved in the upregulation of IL-37 induced by TWG. This study indicates a new possible mechanism for TWG to curb inflammation.
