# Supplementary figures and images for: Tripterygium wilfordii Glycosides Upregulate the New Anti-Inflammatory Cytokine IL-37 through ERK1/2 and p38 MAPK Signal Pathways
Source: Evid Based Complement Alternat Med. 2017 Dec 18;2017:9148523. doi: 10.1155/2017/9148523 (PMC5748296; doi:10.1155/2017/9148523)

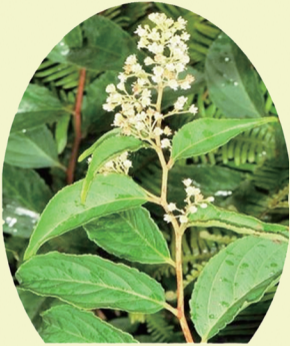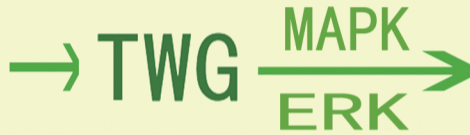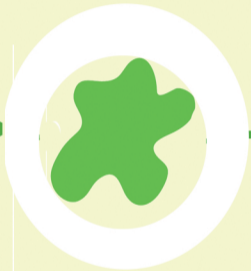

IL-37

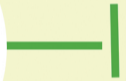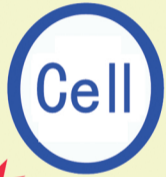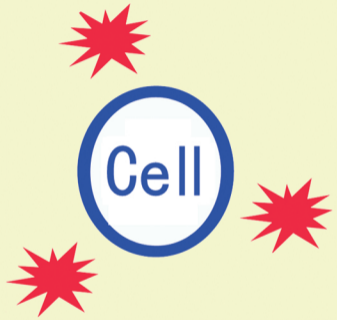

Inflammatory molecules

Supplement: Supplementary Materials — IL-37 is one of the few anti-inflammatory cytokines. TWG, a stable glycoside extracted from Tripterygium wilfordii Hook F (TwHF), is a powerful anti-inflammatory and immune modulatory drug. In this study, it is found that TWG could upregulate IL-37, and ERK1/2 and p38 MAPK pathways might be involved in the upregulation of IL-37 induced by TWG. This study indicates a new possible mechanism for TWG to curb inflammation. [file 9148523.f1.pdf]
